# Global burden of breast cancer and application of patient-reported outcomes in clinical trials: a systematic analysis based on the global burden of disease study 2021 and WHO international clinical trial register database

**DOI:** 10.3389/fonc.2025.1557080

**Published:** 2025-06-12

**Authors:** Jiaxin Hao, Yijia Gong, Xiaowen Zhang, Minghong Du, Huan Wang, Guolin Guo, Mengqing Zhou, Tian Tian, Hongguo Rong

**Affiliations:** ^1^ Center for Evidence-Based Chinese Medicine, Beijing University of Chinese Medicine, Beijing, China; ^2^ Key Laboratory of Chinese Internal Medicine of Ministry of Education, Dongzhimen Hospital, Beijing University of Chinese Medicine, Beijing, China; ^3^ Institute for Excellence in Evidence-Based Chinese Medicine, Beijing University of Chinese Medicine, Beijing, China; ^4^ School of Traditional Chinese Medicine, Beijing University of Chinese Medicine, Beijing, China

**Keywords:** breast cancer, global burden of disease, patient-reported outcomes, clinical trials, systematic analysis, quality of life

## Abstract

**Background:**

Breast cancer is one of the most prevalent tumors worldwide, significantly compromising the survival and quality of life of patients. This study aims to evaluate the global burden and the application of patient-reported outcomes (PROs) in clinical trials of breast cancer.

**Methods:**

Data of breast cancer burden is extracted from the Global Burden of Disease Study (GBD) 2021 database. This study analyzes geographic patterns, temporal trends and age patterns of female breast cancer disease burden globally and explored the association between age standardised rates for disability adjusted life years (ASDR) of female breast cancer and sociodemographic index (SDI). The interventional clinical trials of breast cancer are selected in the WHO International Clinical Trial Register database from January 1, 2010, to December 31, 2022. The application of PROs is classified into three categories: 1) precisely listed PRO instruments as outcomes, 2) mentioned patient subjective feelings without clarifying specified PRO instruments, and 3) not mentioned any PROs as outcomes.

**Results:**

Globally, in 2021 the age standardised rates for point prevalence of female breast cancer per 100000 population was 450.64 (427.02 to 475.96), the age standardised rates for incidence (ASIR) per 100000 population was 46.40 (43.26 to 49.56), and the ASDR per 100000 population was 455.56 (426.64 to 485.30). Compared with 1990, the ASIR of female breast cancer in 2021 had increased while the ASDR had decreased globally. Trials involving PROs only account for 37.87% (3968/10478). The Visual Analog Scale and Cancer Quality of Life Questionnaire-Core 30 are the most common instruments in these trials.

**Conclusions:**

The disease burden of breast cancer is severe and varied worldwide while the application of PROs in clinical trials remains noteworthy. Increasing population awareness about policy for breast cancer care and the application of specific PRO instruments is warranted to reduce the future burden of disease.

## 1 Introduction

According to the International Agency for Research on Cancer (IARC), breast cancer is a significant global health challenge, ranking as the first leading cause of cancer-related deaths worldwide for the year 2020 (1, 2). Globally, the incidence of breast cancer is still increasing, with 4.4 million cases anticipated by 2070 (3). Female breast cancer led in most countries in terms of cancer incidence and death rates in 2020, accounting for approximately 24.5% of all cancer cases and 15.5% of cancer deaths in women (2). The burden of breast cancer has a significant impact on survival rates and quality of life, not limited to its high incidence.

Female breast cancer affects every country, but significant geographical variations exist worldwide (1). Despite the continuous improvement of medical services, female breast cancer is still diagnosed in the advanced stages in the countries without adequate medical resources and appropriate treatment. Studying the regional differences in spatial-temporal patterns (incidence, mortality, prevalence) of female breast cancer can be helpful to determine the underlying causes of these differences and offer guidance for public health policy interventions locally and regionally. Most previous studies concerning female breast cancer incidence and mortality in various regions were insufficient and the reports based on the Global Burden of Disease (GBD) 2021 lacked. All of the required information is connected with the local cancer epidemiology, which can be provided accurately by the GBD 2021. Given the substantial burden of female breast cancer, the incorporation of patient-reported outcomes (PROs) in clinical trials can offer distinct advantages, including improved patient-clinician communication, enhanced health-related quality of life, and financial savings from reduced healthcare utilization.

With the increasing burden of female breast cancer, the importance of utilizing PROs as one of the primary or secondary end points became more prominent (4). PROs play a significant role in informing patient-centered care, enhancing clinical decisions, and improving health policy (5–7), reported by patients themselves and not explained by clinicians or other individuals (8). Data collected using PROs is more comprehensive than conventional medical records and has better sensitivity. The present study extracted data on female breast cancer burden from the GBD 2021, while the registration information of clinical trials was retrieved from the WHO international clinical trial register database. This study aims to facilitate interpretation of female breast cancer burden and analyze the application of PROs in clinical trials for breast cancer, offering insights for future directions.

## 2 Methods

### 2.1 Data sources and collection

The disease burden data for female breast cancer is acquired from the GBD 2021 database. The global burden of disease is a resource that could comprehensively and comparably measure the epidemiological level and trends all over the world. Data on incidence, prevalence, death, disability adjusted life years (DALYs), and percentage change between 1990 and 2021 are retrieved in different age, region, and country groups. Geographical factors are used to identify the 21 GBD regions. The data covered 204 countries/territories globally. The study includes interventional clinical trials of breast cancer which recruited participants aged 18 and older. The clinical trials of breast cancer are selected in the WHO International Clinical Trial Register database from January 1, 2010, to December 31, 2022. The information used to evaluate the conditions and characteristics of the clinical experiments was as follows: 1) basic information, containing the number of registrations, registration date, formal title and country, 2) key information, including PRO instrument, target size, age and gender of participants, and 3) characteristic information, such as primary sponsor and study phase.

### 2.2 Data classification

To estimate the distribution of the disease across different age groups, the individuals are divided into different age groups. Sociodemographic index (SDI) is calculated based on the per capita national income, the total fertility rate of people under 25 and average educational attainment (age ≥ 15 years) (9). According to SDI values, countries and territories are divided into five ranges, named: low SDI, low-middle SDI, middle SDI, high- middle SDI and high SDI region. Countries with higher levels of SDI have higher levels of development (10). The US Food and Drug Administration defined PRO instruments in 2009 as any report of a patient’s health status obtained directly from the patient, without the involvement of a clinician or anyone else to interpret the patient’s response (11). In our study, trials were classified into three categories according to 1) explicitly specified PROs (trial registration information mentioned the use of specified PRO instruments), 2) implicitly specified PROs (trial registration information mentioned the application of PRO instruments, but PRO instruments were not specified), and 3) did not mention any PROs as outcomes.

### 2.3 Statistical analysis

The current study used Joinpoint regression models, smoothing splines models, and annual percent change (APC) to estimate female breast cancer burden. Data concerned with the characteristics of the included studies was extracted independently by two authors using pre-designed data extraction tables. The intervention model, masking method, study phase, participant age and sex, and sample size were summarized. Trials involving PROs were defined as those that used PRO instruments as primary or secondary outcomes (8). This study summarized the most frequently used PRO instruments of included trials, which are used in primary, secondary or co-primary outcomes. The study limited quantitative analysis to items that mentioned the names of PRO instruments to have a more comprehensive understanding of the most often utilized evaluation instruments.

Joinpoint regression models are applied to demonstrate the time trends in the age standardised incidence rate (ASIR) and age standardised mortality rate (ASMR) for female breast cancer in five SDI regions for the period 1990-2021. A maximum number of seven-line segments were used in these models. APC represents the annual percent change, which is calculated in this study. APC is greater than zero, which proves an increasing trend during this period. APC is less than zero, which proves a decreasing trend during this period (12). In order to ascertain the form of the relationship between the age standardised rates for disability adjusted life years (ASDR) and the SDI for 21 regions and 204 countries/territories, our research employed smoothing splines models. The GHDx query tool was utilized to obtain SDI data gathered for 21 regions and 204 countries/territories globally between 1990 and 2021. R software version 4.3.2 and Python 3.8 were used to map the result of female breast cancer burden.

## 3 Results

### 3.1 The burden of female breast cancer at the global level

**Table 1** shows the prevalent cases of female breast cancer was 20.32 million (95% uncertainty interval 19.25 million to 21.45 million), with an age standardised rate prevalence per 100000 population of 450.64 (95% uncertainty interval 427.02 to 475.96), which changed from 1990 (0.11%, uncertainty interval 0.02% to 0.20% per 100000 population). Globally, the number of incident cases of female breast cancer was 2.08 million in 2021 (95% uncertainty interval 1.94 million to 2.23 million), with an age standardised point incidence per 100000 population of 46.40 (95% uncertainty interval 43.26 to 49.56), which changed from 1990 (0.16%, 95% uncertainty interval 0.09% to 0.24%) per 100000. Globally, the number of DALYs due to female breast cancer in 2021 was 20.25 million (95% uncertainty interval 18.96 million to 21.57 million), with an age standardised rate per 100000 population of 455.56 (95% uncertainty interval 426.64 to 485.30), which changed from 1990 to 2021 (-0.10%, 95% uncertainty interval -0.15% to -0.03% per 100000 population).

**Table 1:** Prevalence, incidence, and DALYs of female breast cancer in 2021, and percentage change of ASR per 100000 population between 1990 and 2021.

Regions	Prevalence			Incidence			DALYs		
	ASRs per 100000 population (95%UI)	% change in ASRs per 100000 population (95%UI)	No (95%UI)	ASRs per 100000 population (95%UI)	% change in ASRs per 100000 population (95%UI)	No (95%UI)	ASRs per 100000 population (95%UI)	% change in ASRs per 100000 population (95%UI)	No (95%UI)
Global	450.64 (427.02 to 475.96)	0.11 (0.02 to 0.20)	20323179.27 (19248043.55 to 21451411.54)	46.40 (43.26 to 49.56)	0.16 (0.09 to 0.24)	2082737.02 (1940351.20 to 2225082.58)	455.56 (426.64 to 485.30)	-0.10 (-0.15 to -0.03)	20254801.61 (18963375.54 to 21574428.57)
High SDI	828.58 (791.29 to 860.87)	0.00 (-0.06 to 0.06)	8041601.99 (7599008.96 to 8420079.03)	77.08 (79.93 to 71.83)	-0.03 (-0.06 to 0)	724293.16 (661769.29 to 756432.83)	467.07 (437.15 to 493.59)	-0.35 (-0.37 to -0.33)	4323106.23 (3982603.38 to 4606054.21)
High-middle SDI	501.52 (460.59 to 554.38)	0.28 (0.14 to 0.44)	5042408.42 (4645487.75 to 5543064.84)	51.05 (46.06 to 57.18)	0.31 (0.18 to 0.48)	506490.94 (457358.82 to 565099.76)	424.81 (387.63 to 471.98)	-0.21 (-0.28 to -0.12)	4190122.59 (3830405.58 to 4646696.35)
Middle SDI	328.73 (300.69 to 360.45)	0.81 (0.61 to 1.03)	4780714.02 (4364641.61 to 5246604.07)	37.16 (33.42 to 41.13)	0.81 (0.61 to 1.05)	539058.06 (484448.46 to 597125.95)	415.76 (375.54 to 461.93)	0.12 (0.00 to 0.25)	6040957.75 (5448280.24 to 6714089.23)
Low-middle SDI	224.70 (206.59 to 241.85)	0.85 (0.64 to 1.09)	1880826.03 (1726303.32 to 2021532.46)	28.29 (25.52 to 30.93)	0.92 (0.71 to 1.17)	235445.69 (212856.91 to 257145.85)	478.07 (430.12 to 525.81)	0.44 (0.27 to 0.65)	4061107.51 (3659393.83 to 4465154.90)
Low SDI	173.21 (155.67 to 190.48)	0.51 (0.28 to 0.77)	557520.44 (498270.22 to 618791.07)	24.09 (21.34 to 26.87)	0.54 (0.30 to 0.85)	75293.87 (66491.48 to 84809.81)	488.24 (431.45 to 547.92)	0.28 (0.06 to 0.56)	1616943.25 (1414471.96 to 1832517.40)
WHO Eastern Mediterranean Region	407.38 (366.37 to 445.97)	1.11 (0.83 to 1.40)	1100998.12 (986510.66 to 1214384.46)	46.87 (41.13 to 52.36)	1.29 (0.96 to 1.65)	127387.68 (111422.99 to 142737.01)	551.51 (471.74 to 646.59)	0.46 (0.21 to 0.74)	1565028.69 (1326813.17 to 1842168.96)
WHO South-East Asia Region	212.38 (187.05 to 241.51 to)	0.84 (0.62 to 1.10)	2174859.23 (1911876.52 to 2474514.62)	25.80 (22.16 to 30.12)	0.85 (0.61 to 1.16)	263253.30 (226558.27 to 307025.38)	432.83 (371.65 to 506.08)	0.33 (0.16 to 0.56)	4470963.22 (3836667.08 to 5219937.53)
WHO African Region	237.49 (210.26 to 265.97)	0.59 (0.37 to 0.84)	785599.68 (684717.95 to 891000.69)	32.87 (28.61 to 37.42)	0.60 (0.36 to 0.92)	105508.46 (90272.47 to 121239.56)	617.55 (528.43 to 711.45)	0.32 (0.09 to 0.61)	2103241.70 (1780454.70 to 2432183.98)
WHO Region of the Americas	694.29 (660.90 to 726.24)	-0.14 (-0.19 to -0.09)	4825584.89 (4579846.00 to 5062610.24)	69.80 (65.38 to 73.15)	-0.14 (-0.18 to -0.11)	478792.04 (444691.09 to 502368.09)	516.39 (486.29 to 548.31)	-0.28 (-0.31 to -0.25)	3467034.32 (3259631.11 to 3683136.20)
WHO European Region	701.18 (670.91 to 731.91)	0.12 (0.03 to 0.19)	5735177.67 (5460495.94 to 6005665.71)	67.50 (63.73 to 70.63)	0.11 (0.06 to 0.15)	539075.28 (498431.95 to 566174.80)	522.21 (489.35 to 555.60)	-0.28 (-0.32 to -0.25)	4116783.15 (3828588.53 to 4389014.36)
WHO Western Pacific Region	396.87 (343.84 to 460.14)	0.76 (0.47 to 1.13)	5547050.70 (4833863.32 to 411855.68)	40.16 (33.41 to 47.84)	0.89 (0.51 to 1.36)	553525.86 (462661.48 to 659095.97)	323.75 (273.19 to 384.35)	0.02 (-0.18 to 0.29)	4404246.85 (3732051.82 to 5227847.64)

### 3.2 Burden of female breast cancer in five SDI regions

**Table 1** presents that the highest age standardised point prevalence of female breast cancer per 100000 population in 2021 was in the high SDI region [828.58 (95% uncertainty interval 791.29 to 860.87)], while the lowest was in the low SDI region [173.21 (95% uncertainty interval 155.67 to 190.48)]. In 2021, the highest ASIR of female breast cancer per 100000 population was the high SDI region [77.08 (95% uncertainty interval 71.83 to 79.93)], while the lowest was the low SDI region [24.09 (21.34 to 26.87)]. In 2021, the highest ASDR of female breast cancer per 100000 population was the low SDI region [488.24 (95% uncertainty interval 431.45 to 547.92)], while the lowest was the middle SDI region [415.76 (375.54 to 461.93)].

### 3.3 Burden of female breast cancer in six WHO regions

**Table 1** shows that the highest age standardised point prevalence of female breast cancer per 100000 population was in the WHO European Region [701.18 (95% uncertainty interval 670.91 to 731.91)], while the lowest was in the WHO South-East Asia Region [212.38 (95% uncertainty interval 187.05 to 241.51)] by 2021. Except for the WHO Region of the Americas, other regions were on the increase. The highest ASIR of female breast cancer per 100000 population was in the WHO Region of the Americas [69.80 (95% uncertainty interval 65.38 to 73.15)], while the lowest was in the WHO South-East Asia Region [26.0 (95% uncertainty interval 22.0 to 30.4)] by 2021. Except for the WHO Region of the Americas, other WHO regions were on the increase. The highest ASDR of female breast cancer per 100000 population was in the WHO African Region [617.55 (95% uncertainty interval 528.43 to 711.45)], while the lowest was in the WHO Western Pacific Region [323.75 (95% uncertainty interval 273.19 to 384.35)] by 2021. All regions, except for the WHO Region of the Americas and the WHO European Region, showed an upward trend.

### 3.4 The burden of female breast cancer at the national level

The countries with the highest ASIR estimated per 100000 population in 2021 were the Principality of Monaco [163.82 (120.64 to 221.94)], United Arab Emirates [131.81 (97.03 to 174.18)], Qatar [125.28 (92.03 to 174.18)], whereas Mongolia [11.37 (8.74 to 14.12)], Niger [13.02 (8.94 to 18.03)], The Gambia [15.07 (10.97 to 20.04)] had the lowest rates (**Supplementary eFigure 1** in the (**Supplementary Material**). The countries with the highest age standardised point prevalence estimates per 100000 population in 2021 were Principality of Monaco [1689.45(1348.34 to 2153.73)], United Arab Emirates [1235.12 (979.40 to 1550.47)], Qatar [1145.79 (894.26 to 1457.43)], whereas Niger [91.26 (67.87 to 119.97)], Mongolia [99.65 (83.12 to 118.37)], The Gambia [(109.59 (84.85 to 140.94)] had the lowest rates.

### 3.5 Time trends of female breast cancer incidence and mortality in five SDI regions

**Figure 1:**
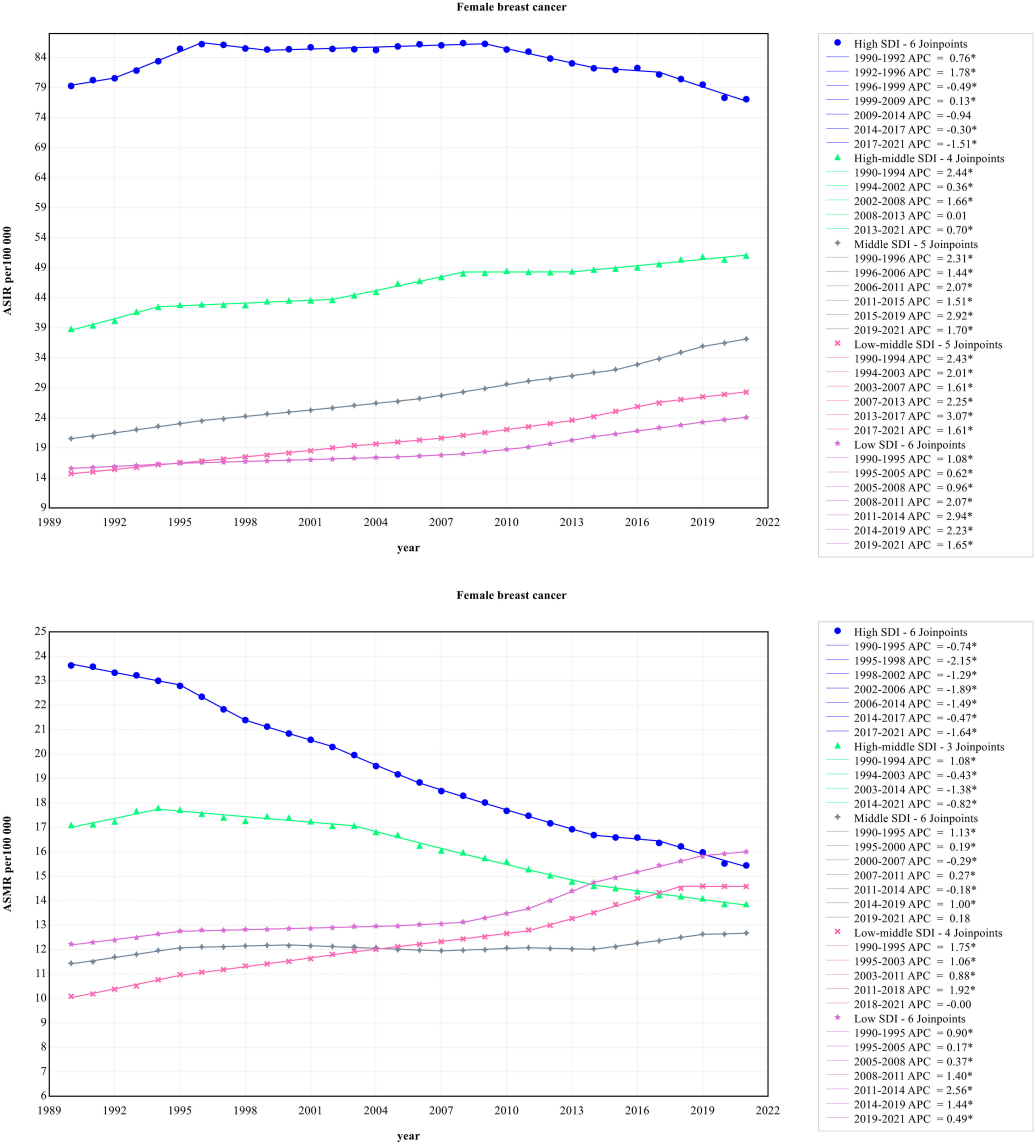
Trends in ASIR and ASMR of female breast cancer by five SDI regions.

**Figure 1** presents the time trends of female breast cancer incidence that were significantly different in five SDI regions. From the perspective of incidence, the ASIR of the high SDI region was obviously higher than other SDI regions. From 1990 to 2021, the ASIR of the high SDI region remained high, while the other four SDI regions showed a small growth trend. From 2009 to 2021, although the high SDI region had the highest ASIR, a steady downward trend was observed. The high SDI region had the highest ASMR of female breast cancer, meanwhile with a sharp decrease throughout the study period. The ASMR of female breast cancer in the middle SDI region slowly increased from 1990 to 1994 and decreased between 1995 and 2021. However, other SDI regions showed an andante upward trend.

### 3.6 Age patterns of female breast cancer in five SDI regions

**Figure 2:**
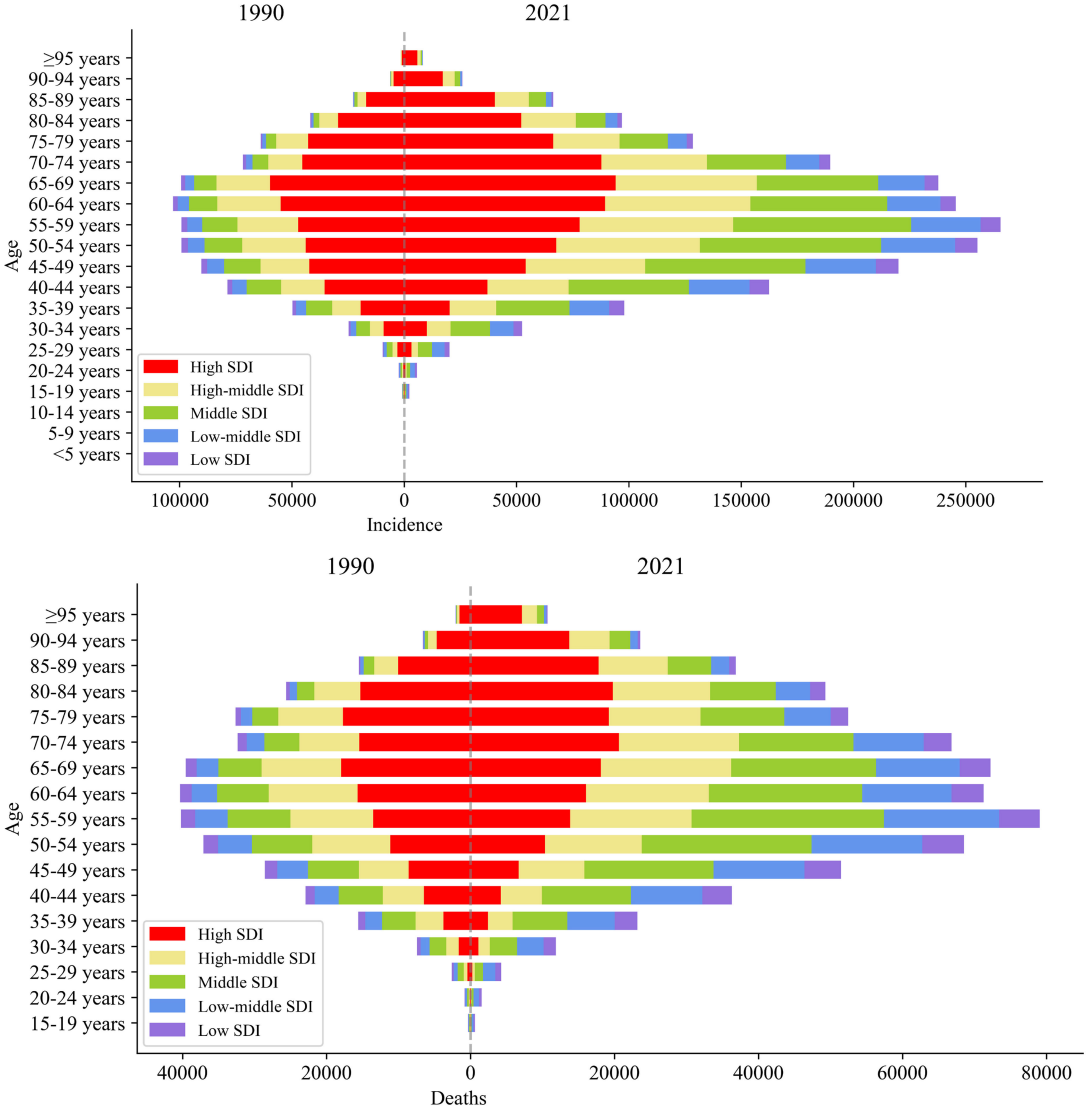
Incidence and deaths of female breast cancer by SDI and age, 1990 and 2021.

**Figure 2** illustrates that age had a significant impact on the incidence and mortality of female breast cancer. In 1990 the number of incidence cases of female breast cancer increased with age up to 60-64 years, then decreased with older age. In 2021 the peak of female breast cancer incidence was found in the 55-59 age group, which was in a younger age group than that in 1990. Similarly, in 2021 the mortality increased with age and peaked at the 55-59 and 65-69 age groups for females, then decreased with older age. Compared with 1990, the mortality in 2021 declined generally with ages up to 65-69 years, especially in the high SDI regions.

### 3.7 Observed burden of female breast cancer compared with expected by SDI

**Figure 3:**
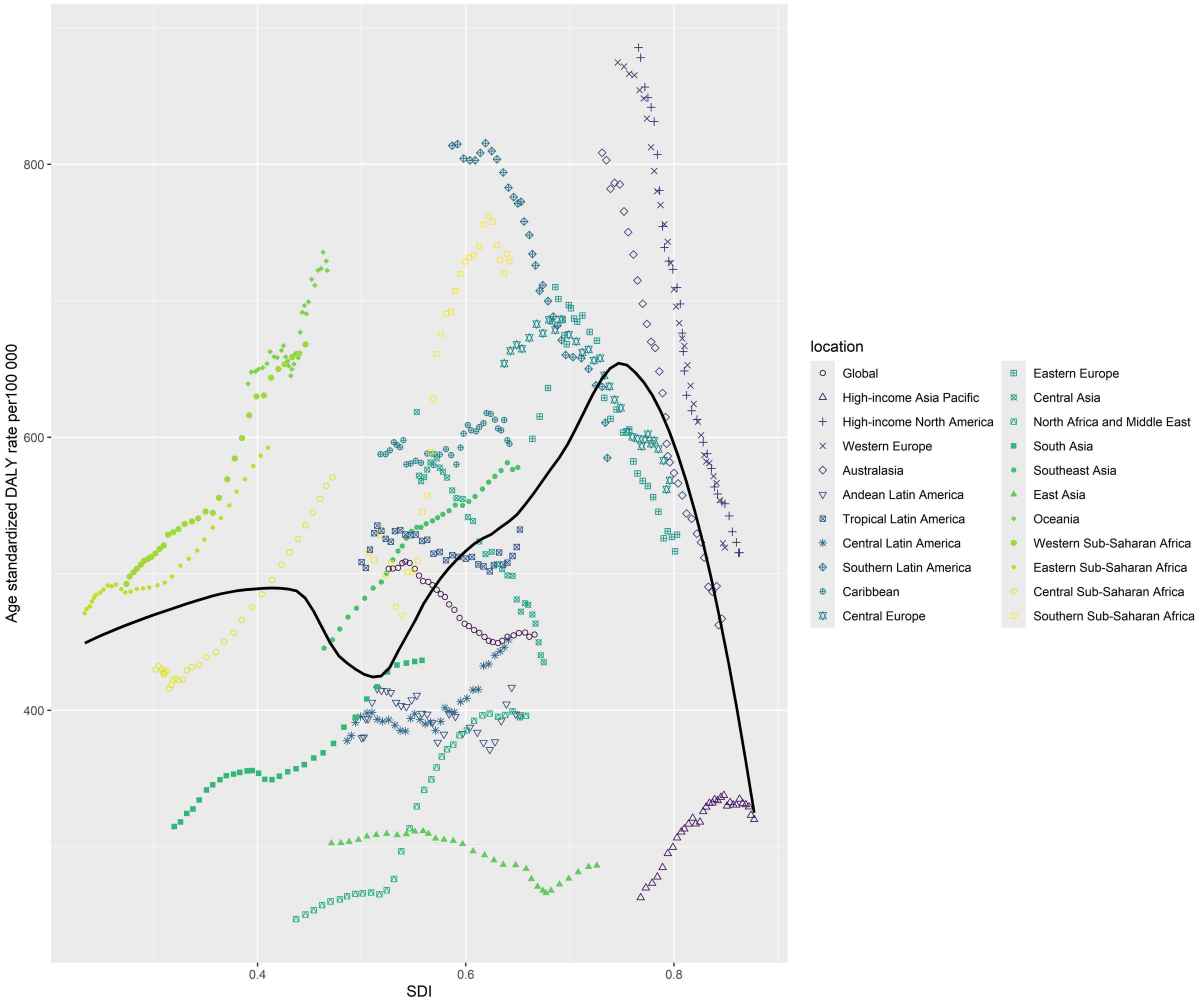
Age standardised DALY rate from female breast cancer per 100 000 population for 21 Global Burden of Disease regions by sociodemographic index, 1990-2021.

**Figure 3** demonstrates a time trend of the relationship for each of the countries and regions, with each colored line denoting a particular year. Based on data from all nations from 1990 to 2021, the average expected association between SDI and DALYs for breast cancer was represented by the black line with a 95% confidence range. DALYs were calculated by summing YLDs (years lived with disability) and YLLs (years of life lost). One DALY corresponded to one healthy year lost (13).

Overall, a positive association was observed between the ASDR of female breast cancer and SDI across the GBD regions of which SDI was under 0.75. At the regional level, the observed burden was higher than expected given the sociodemographic index for high-income North America, Western Europe, Western Sub-Saharan Africa, Eastern Sub-Saharan Africa and Southern Sub-Saharan Africa.

**Figure 4:**
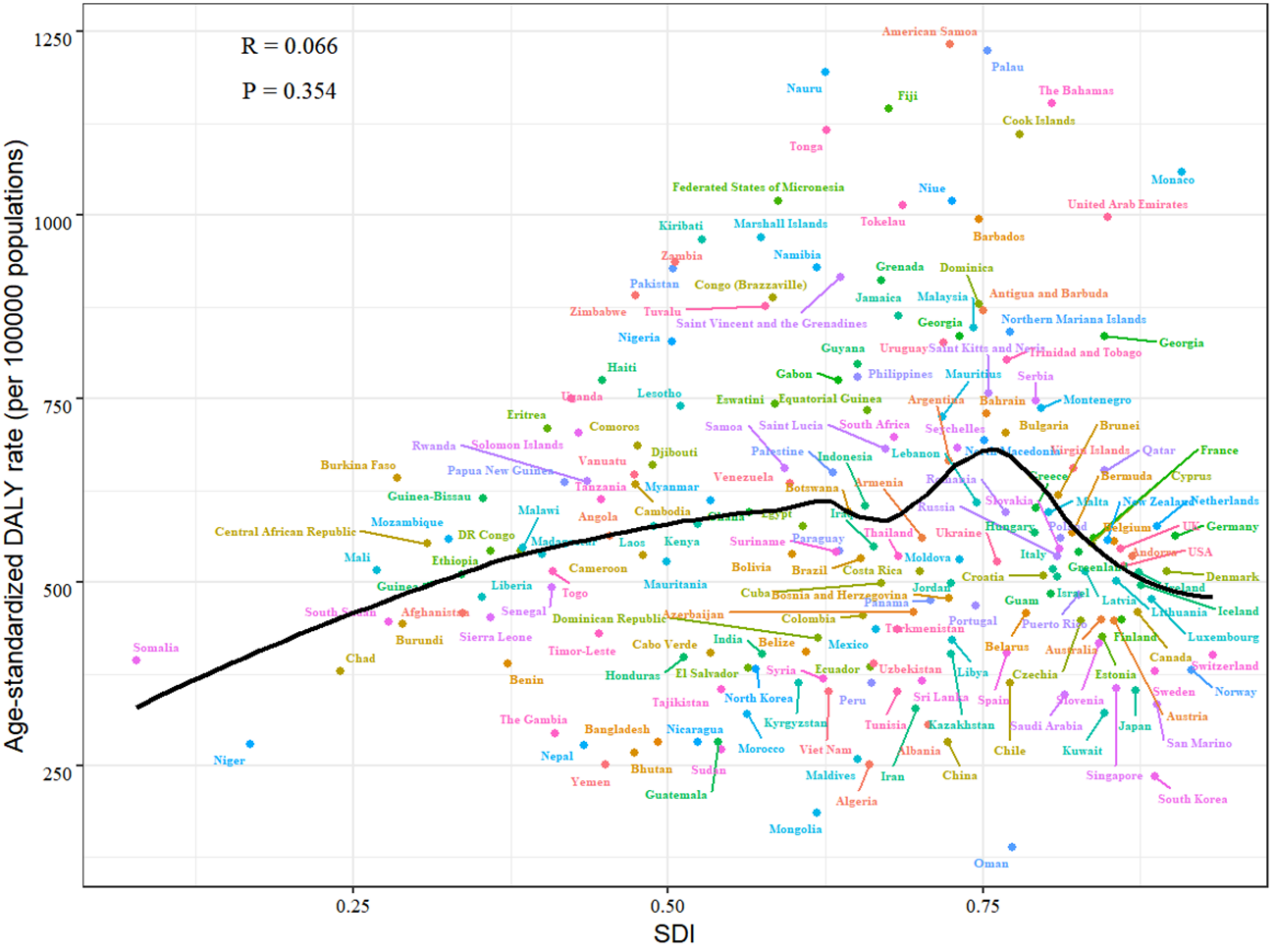
Age standardised DALY rate from female breast cancer per 100 000 population by countries and SDI, 2021.

**Figure 4** illustrates the high ASDR of female breast cancer was not limited to developed countries. In developing countries such as Malaysia, Nigeria and Jamaica, the ASDR was much higher than the expected level based on SDI. Whereas in developed countries such as Japan, Australia and South Korea, the ASDR was much lower than expected.

### 3.8 Trial characteristics

The sex characteristics of the included trials are presented in **Supplementary eFigure 2** in the **Supplementary Material**. Our study identified 11043 interventional studies conducted from 2010 to 2022 all over the world. Our study excludes 565 trials, including 110 duplicates, 428 clinical trials including children, and 27 outcomes not filled or reported. A total of 10478 eligible trials are identified for analysis. Among the 10478 included trials, 3968 trials mention the use of PROs. Among the 3968 trials involving PROs, 997 trials mention the use of PROs but instruments are not specified, and 2971 trials mentioned the use of PROs and instruments are specified.

A comprehensive overview of eligible trials is presented in **Table 2**. Among the included trials, phase-2 trials [2562 (24.45%)] are the most common, followed by phase-3 trials [(1161 (11.08%))]. Of the 3968 trials involving PROs, phase 2 trials [560 (14.11%)] were still the most common, followed by phase 3 [494 (12.45%)] trials (**Table 1**). Among the included trials, most of the experimental subjects were over 18 years old [9648 (92.08%)], and only a few of them were over 65 years old [122 (1.16%)]. Of the 3968 trials involving PROs, most of the trial participants are still over 18 years old [3608 (90.93%)], and only a few of them are over 65 years old [54 (1.36%)]. Among the included trials, more than 60.0% of the studies chose women as the research objects, while 30.65% had no limitation on sex, and only 0.45% of trials chose men as the research participants. Of the 3968 trials involving PROs, more than 65.00% of the trials chose women as the research participants, while 23.06% had no limitation on sex, and only 0.43% of trials chose men as the research participants. Most primary sponsors were located in the WHO Region of the Americas, followed by the WHO European Region and the WHO Western Pacific Region. The other WHO regions, including the WHO South-East Asia Region, the WHO Eastern Mediterranean Region and the WHO African Region accounted for less than 11% of the trial locations (**Table 1**). Similar findings were found when only PRO-related studies were considered, with more than 80% of primary sponsors coming from the WHO Region of the Americas, the WHO European Region and the WHO Western Pacific Region. Meanwhile fewer than 20% of sponsors came from the WHO Eastern Mediterranean Region, the WHO South-East Asia Region and the WHO African Region.

**Table 2:** Characteristics of all trials and trials including PROs.

Characteristics	Classifications	Trials	Total, No. (%)
			PRO trials
No.		10478	3968 (37.87)
Method allocation	Randomized	5624 (53.68)	2989 (75.33)
	Non-randomized	1989 (18.98)	379 (9.55)
	NA	2865 (27.34)	600 (15.12)
Intervention Model	Single arm	3694 (35.25)	816 (20.56)
	Parallel	5073 (48.42)	2634 (66.38)
	Crossover	244 (2.33)	89 (2.24)
	NA	524 (5.00)	153 (3.86)
	Othera	943 (9.00)	276 (6.96)
Masking	Single blind	713 (6.80)	479 (12.07)
	Double blind	906 (8.65)	505 (12.73)
	Triple blind	266 (2.54)	164 (4.13)
	Quadruple	198 (1.89)	101 (2.55)
	Open label	7283 (69.51)	2245 (56.57)
	Unclear	1112 (10.61)	474 (11.95)
Sample size	≤ 200	8117 (77.46)	2979 (75.07)
	201-400	1059 (10.11)	458 (11.54)
	401-1000	674 (6.43)	271 (6.83)
	1000+	404 (3.86)	176 (4.44)
	NA	224 (2.14)	84 (2.12)
Recruitment status	Recruiting	2960 (28.25)	1177 (30.00)
	Not yet recruiting	7098 (67.74)	2649 (69.77)
	NA	26 (0.25)	1 (0.03)
	Otherb	394 (3.76)	8 (0.20)
Study phase	Early stage^c^	1342 (12.81)	220 (5.54)
	2	2562 (24.45)	560 (14.11)
	3	1161 (11.08)	494 (12.45)
	4	425 (4.06)	152 (3.83)
	5	1 (0.01)	0 (0.00)
	NA	4159(39.69)	2369 (59.71)
	Other^d^	828 (7.90)	173 (4.36)
age	18-no limit	9648 (92.08)	3608 (90.93)
	Over 65	122 (1.16)	54 (1.36)
	Unclear	708 (6.76)	306 (7.71)
Sex	Female	6600 (62.99)	2764 (69.66)
	Male	47 (0.45)	17 (0.43)
	Male and female	3211 (30.65)	915 (23.06)
	NA	620 (5.92)	272 (6.85)
WHO Region	WHO African Region	23 (0.22)	11 (0.28)
	WHO Region of the Americas	3637 (34.73)	1193 (30.10)
	WHO Eastern Mediterranean Region	515 (4.92)	334 (8.42)
	WHO European Region	2873 (27.42)	1221 (30.80)
	WHO South-East Asia Region	559 (5.33)	257 (6.48)
	WHO Western Pacific Region	2869 (27.38)	949 (23.92)

^a^Cluster randomized, cross-sectional, dose comparison, factorial, historical control, N of 1 Trial, pragmatic two-arm, quasi-randomized controlled, sequential assignment, multicentre randomized controlled trial.

^b^Authorised, authorised-recruitment may be ongoing or finished, completed, withdrawn.

^c^Early phase trials include clinical phase 0, phase 1 trials and clinical pre-test.

^d^Diagnostic new technique clinical study and trials involving multiple stages.

**Figure 5:**
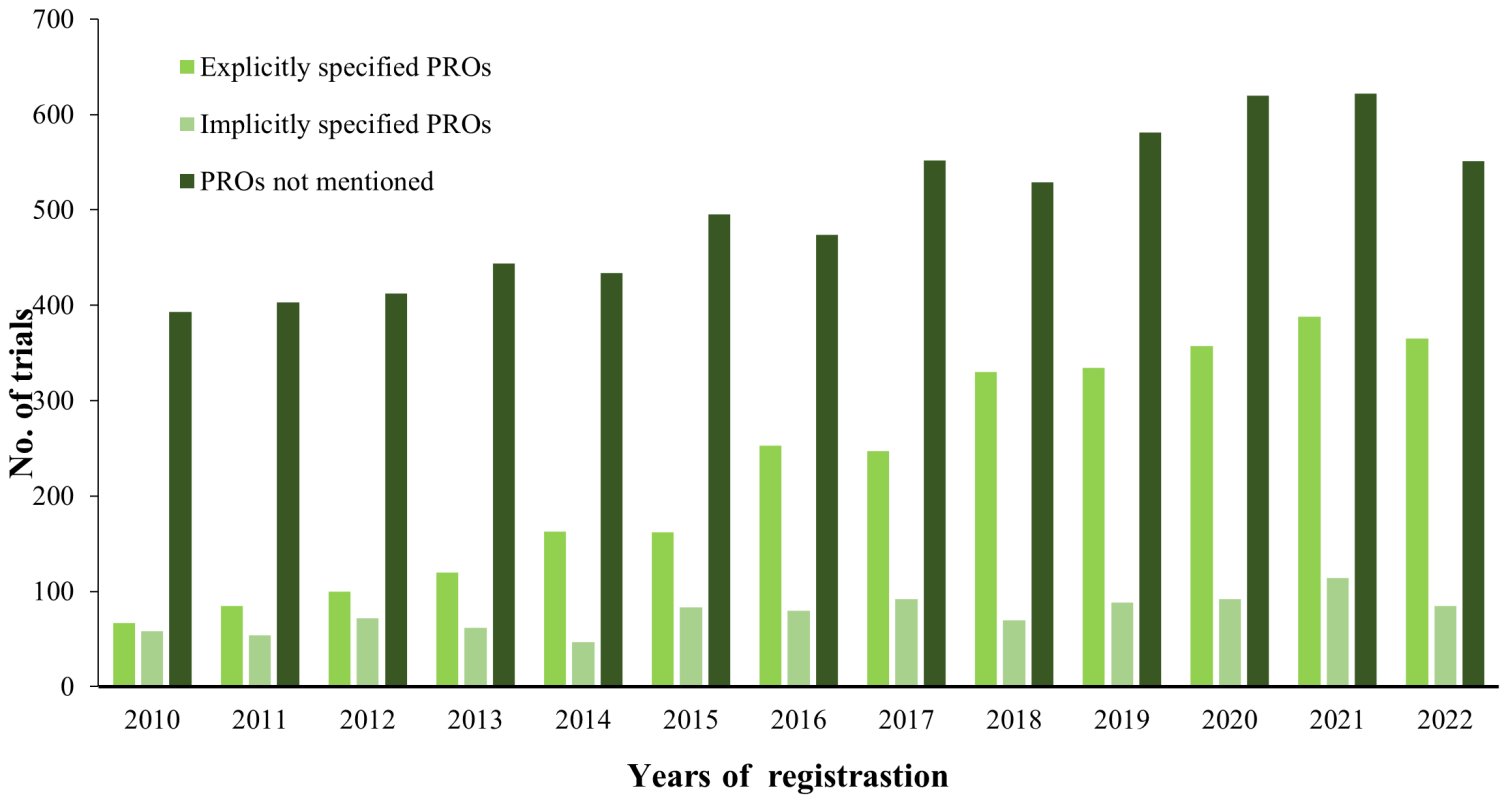
Number of clinical trials analyzed.

**Figure 5** illustrates the increase in the number of breast cancer clinical registration trials from 2010 to 2022 and demonstrates the proportion of trials that listed PROs as the outcome among the breast cancer trials. Of the 10478 trials, 28.4% (n = 2971) report specific PRO instruments, 9.5% (n = 997) are trials that use a vague PRO description, and 62.1% (n=6510) are trials that not use PROs. PRO-related trials are also on the increase, particularly the trials with explicitly specified PROs.

### 3.9 Global distribution of trials involving PROs

There are among different countries. The distribution of primary sponsors among the different countries of trials involving PROs is shown in **Supplementary eFigure 3** in the **Supplementary Material**. Among the trials involving PROs, most primary sponsors are seated in Europe, followed by Asia, North America and Africa. The other regions, including South America, Oceania and Antarctica accounted for 0%–6.9% (**Table 2**). The top ten countries are the United States, China, Iran, India, Japan, France, Canada, United Kingdom, Australia and Germany. Meanwhile United States, Canada and Australia were also countries with high incidence and prevalence of female breast cancer.

### 3.10 PRO instruments used in clinical trials

To summarize the overall application of PROs, our study collected the specific PRO instruments applied in trials that explicitly mention the PRO instruments as primary and/or secondary outcomes (**Table 3**). In the trials that applied PRO instruments as primary outcomes, VAS was the most frequently used PRO instrument. Moreover, EORTC QLQ-C30 was the most frequently used PRO instrument in the trials that applied PRO instruments as secondary outcomes. The frequently utilized PRO instruments applied as co-primary outcomes were EORTC QLQ-C30, VAS, EORTC QLQ-BR23, FACT-B, HADS, NRS, EQ-5D-5L, BREAST-Q, PSQI and BPI. Although PRO instruments were used in different outcomes, VAS, EORTC QLQ-C30, FACT-B, HADS, NRS and EORTC QLQ-BR23 were still used frequently. EORTC QLQ-C30, EORTC QLQ-BR23 and EQ-5D-5L are scales to assess health-related quality of life. VAS, NRS and BPI are scales to evaluate pain. HADS and PSQI are scales to measure symptoms of mental health. FACT-G, FACIT-F and FACT-B are scales to analyze function.

**Table 3:** Frequency of PRO instruments utilized as primary outcomes, secondary outcomes and co-primary outcomes.

No. of PROs	Primary outcome	Secondary outcome	Co-primary outcome
1	VAS (179)	EORTC QLQ-C30 (519)	EORTC QLQ-C30 (643)
2	EORTC QLQ-C30 (121)	VAS (222)	VAS (410)
3	FACT-B (71)	EORTC QLQ-BR23 (241)	EORTC QLQ-BR23 (312)
4	NRS (54)	FACT-B (216)	FACT-B (229)
5	HADS (49)	HADS (169)	HADS (207)
6	BPI (41)	EQ-5D-5L (108)	NRS (159)
7	EORTC QLQ-BR23 (53)	NRS (108)	EQ-5D-5L (129)
8	PSQI (34)	BREAST-Q (73)	BREAST-Q (99)
9	FACIT-F (32)	SF-36 (63)	PSQI (97)
10	SF-36(29)	FACT-G (59)	BPI (97)

VAS, Visual Analogue Scale; EORTC QLQ-C30, European Organization for Research and Treatment of Cancer Quality of Life Questionnaire-Core 30; FACT-B, Functional Assessment of Cancer Therapy-Breast; NRS, Numeric Rating Scale; HADS, Hospital Anxiety and Depression Scale; BPI, Brief Pain Inventory; EORTC QLQ-BR23, European Organization for Research and Treatment of Cancer Quality of Life Questionnaire-Breast Cancer module 23; PSQI, Pittsburgh Sleep Quality Index; FACIT-F, Functional Assessment of Chronic Illness Therapy - Fatigue; SF-36, SF-36 Health Survey; EQ-5D-5L, European Quality of Life-5 Dimension-5 level scale; BREAST-Q, BREAST-Q; FACT-G, Functional Assessment of Cancer Therapy-General.

**Figure 6:**
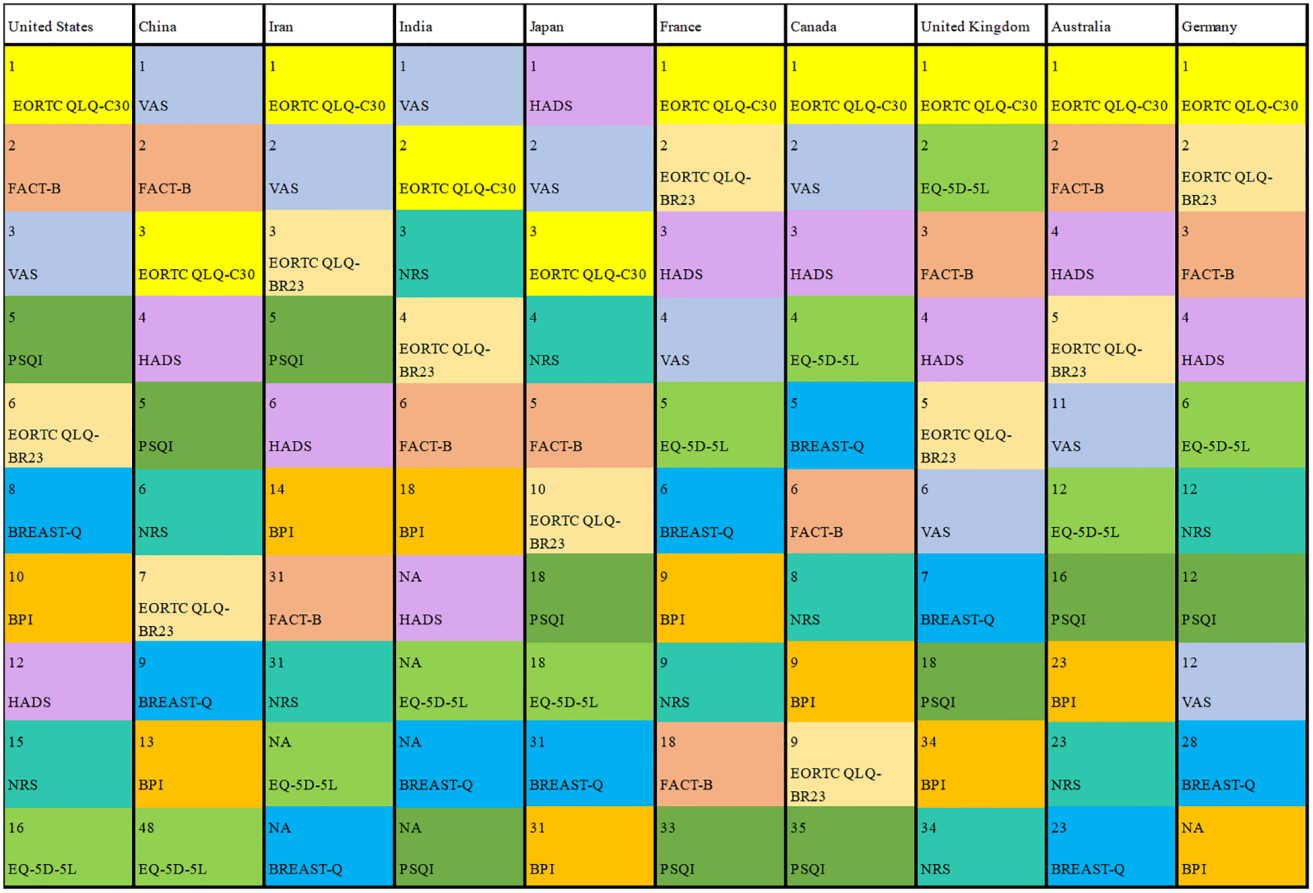
Rankings of frequency of the most commonly applied PRO instruments.

**Figure 6** illustrates the frequency of the top ten used PRO instruments of co-primary outcomes in the US, China, Iran, India, Japan, France, Canada, the UK, Australia and Germany. Different colors represented different instruments while the number represents the ranking of the frequency of use of the instruments in this country. EORTC QLQ-C30 was most frequently applied except for China, India, and Japan. In these countries, health-related quality of life was the main evaluation index of clinical trials involving PROs. VAS was the most frequently used instrument in China and India. HADS was the most frequently used instrument in Japan, which was used to evaluate patients’ anxiety and depression. It can be indicated that anxiety and depression of breast cancer patients are the main indices of clinical trials involving PROs of breast cancer in Japan. BREAST-Q was not used in India and Iran, which reflected less evaluation of satisfaction among patients undergoing breast surgery. HADS and PSQI were out of use in India, which reflected the lack of evaluation of mental health.

## 4 Discussion

To our knowledge, this study is among the first systematic researches to integrate the global burden of disease with the application of PROs in clinical trials for breast cancer. The study had two major findings. Firstly, the burden of female breast cancer is still not alleviated and varied across territories, ages, and SDI levels. Secondly, the study finds that over 60% of the clinical trials neglected subjective burden by patients. Meanwhile, the standard as well as widely used PRO instruments for breast cancer are not enough. It is significant to introduce PROs to the evaluation of breast cancer and assess the burden of breast cancer, which can improve the comprehensiveness, accuracy, and degree of individuation of the evaluation.

According to the GBD 2021, the number of prevalent cases of female breast cancer was 20.32 million with large geographical variations, which is in accordance with the previous studies (14, 15). At the regional level, our study found that the highest age standardised point prevalence estimated in 2021 was in High SDI regions followed by High-middle SDI regions globally, which proved that higher level of SDI was correlated with increased age standardised prevalence rates of female breast cancer. At the national level, Mongolia, Niger and The Gambia had the lowest incidence and prevalence in 2021. This may be related to the national development level, which approved public health policy concerning female breast cancer as fundamental in developing countries (16). It is also significant to develop and adopt the cost-effective screening and therapeutic regimen, which can mitigate the risk factors of female breast cancer in developing countries (17). Meanwhile improving health data of female breast cancer in all countries and regions is strongly suggested for a better evaluation of the Global Burden of Disease.

The incidence estimates of female breast cancer in our study peaks in the middle-aged group, which is also found in other studies (16, 18, 19). The highest incidence was aged 60-64 years in 1990 and aged 55-59 years in 2021. Compared with 1990, the peak of incidence appeared in the younger age groups. The variation may be due to the higher healthcare service level the earlier detection and the change in the population age structure (18, 20). Mammographic screening can reduce mortality of female breast cancer aged 50-74 years (21) and benefit women aged 45-49 years similarly (22). Therefore, it is important to improve the early detection and advanced treatment of women in the middle-aged group, which can increase the survival rate and life expectancy of female breast cancer patients. The development and adoption of cost-effective screening and therapeutic solutions, the mitigation of risk factors, and the establishment of a cancer infrastructure were essential.

Clarity and control of risk factors are two common methods in clinical prevention programs (23). The risk factors for female breast cancer involved different categories, such as various genetic, environmental, lifestyle, demographic and socioeconomic risk factors (24, 25). DALYs and related socioeconomic risk factors had an influence on the incidence and development of female breast cancer around the world. Our study found that the high ASDR of female breast cancer was not limited to regions and territories with high SDI, while the mortality among high SDI regions and high-middle SDI regions declined during 1990-2021. Since 1990, the correlation between socioeconomic development and discrepancy in the global female breast cancer incidence had continued to decline (15). This phenomenon proved the centralization of the disease burden had transformed from developed to undeveloped countries. This change in DALYs from female breast cancer can be attributed to the following factors. Firstly, alcohol consumption was the greatest distributor of breast cancer DALYs and mortality (19, 26). Studies which demonstrated the use of alcohol declined significantly in the past three decades especially in the high and the high middle SDI countries, while increased in most developing countries. Therefore, policy makers are recommended to reject commercial marketing for products especially alcohol, which can raise the risk of breast cancer. Secondly, raising awareness among the general public and improving treatment in high SDI regions can contribute to mortality rates reduction and survival rates improvement, especially Mammographic screening (27). Meanwhile, expanding the scope of early detection efforts for breast cancer in low-income and middle-income countries is recommended under the permission of economic conditions, rather than concentrating just on mammography screening. Equitable access to early diagnosis and treatment is a fundamental need for all individuals to improve their breast cancer survival rates and quality of life in different income regions. Moreover, factors of female breast cancer including but not limited to BMI, contraceptive use, family history and menopause could impact the DALYs in countries with different levels of SDI (28, 29). Our study stressed the importance of breast cancer risk factor education in preventing potential future cases of breast cancer. People should have a healthy lifestyle and address attributable risk factors. Non-genetic, modifiable risk factors should be emphasized to reduce the prevalence of female breast cancer in countries with lower SDI (30).

Our study emphasizes that, despite enormous advances in breast cancer research and treatment over the past three decades, making efforts to an over 40% reduction in breast cancer mortality in some high-income countries, the subjective emotions and suffering of breast cancer patients were still disregarded (1). Many patients with breast cancer suffered from mental health problems as well as none appropriate care even in high-income countries. However, the effects including but not restricted to physical, psychological, social and economic aspects on patients with breast cancer had an impact on patients themselves, their families and society without being fully evaluated. Therefore, enhanced patient decision-making and communication in breast cancer care are encouraged, which can lead to improvements in adherence to therapy, quality of life, and body image. Our research analyzed the application of PROs in clinical trials to improve patient communication and decision-making in breast cancer treatment and reduce the suffering sustained by patients. The findings revealed that among the 10478 eligible trials, only 3968 trial registrations mentioned the use of PROs, which accounted for 37.87%. Among the trials mentioning PROs, 778 applied PROs as primary outcomes, 1930 as secondary outcomes and 1260 as co-primary outcomes. Of the 3968 trials applied PROs, 28.4% (n = 2971) were involved in trials that reported specific PRO instruments. Considering that PROs could appropriately represent patients’ subjective feelings and opinions, they should be emphasized in the study of breast cancer (31). It is of great significance to prioritize patients’ benefits and value patient opinions in clinical trials (32). However, faced with the small proportion of breast cancer-related clinical trials involving PROs, more attention is encouraged to the enormous costs and suffering sustained by patients with breast cancer, which can reduce patients’ suffering and society’s burden of breast cancer.

The number of breast cancer clinical registration trials increased from 2010 to 2022, especially those applied specific PRO instruments. Trials applied PROs of breast cancer are more prevalent in the WHO Region of the Americas followed by the WHO European Region. Conversely, in other regions, especially in the WHO African Region and the WHO South-East Asia Region, the number of clinical trials applied to PROs was conspicuously lower. The reasons were attributed to the following points. Firstly, the economic development level and medical resources of different countries and regions played a significant role in this phenomenon (33). Among the trials involving PROs, most primary sponsors were seated in developed countries including but not limited to the United States, Japan, France, Canada, United Kingdom, Australia and Germany. It was disproportionally distributed with a prominent difference in density between developed and developing countries in health workforce reserve by 2020. It is predicted that the health workforce shortage would improve a lot globally, but the shortage in the WHO African region would continue to be severe. Therefore, developed countries had more medical resources to invest in clinical trials involving PROs of breast cancer (34). Secondly, there existed an intimate relationship between the adoption of PROs in clinical trials of breast cancer and the disease burden. The regions with a great deal of primary sponsors of clinical trials involving PROs, such as the WHO Region of the Americas and the WHO European Region, also had the highest age standardised point prevalence. Clinical Trials on breast cancer in these regions can provide valuable evidence to support collaborative decision-making, claim labeling, medicinal recommendations, and health policy (5). Considering PROs can’t be applied in health resource-constrained regions, it is necessary to apply more simplified instruments and indicators to reveal patients’ subjective pain, including physical, mental and psychological thus meeting the demand of the patients suffering from breast cancer (32). More concentration on treatment considering patients’ subjective pain should be given especially in low-income regions, which had high risk of death concerning breast cancer.

Analyzing the frequency of PRO instruments application in different types of trials, we found the most frequently used questionnaires for breast cancer patients are EORTC-QLQ C30 and VAS. EORTC QLQ-C30 is a proverbial and extensively used representative self-administered questionnaire of breast cancer, which covers multidimensional aspects of health-related quality of life. Meanwhile, breast cancer patients had poor quality of life generally (35, 36). VAS has precision, simplicity, and sensitivity. VAS is accurate, simple and sensitive (37). In addition, a considerable proportion of breast cancer patients suffered from breast cancer pain which was common and heavy but lacked objective evaluation indices (1). Therefore, VAS has become the first choice for pain evaluation in clinical research. There can be variations in the use of PRO instruments concerning breast cancer across different countries. Questionnaires frequently applied in clinical trials concerning breast cancer including BREAST-Q, and HADS scales were not used in India, reflecting the lack of evaluation of mental and patient satisfaction related to breast surgery (38, 39). Our study speculated the reason that the patients in India ignored the treatment-related side effects when customizing the treatment plan, such as the breast shape and psychological conditions of breast cancer patients after an operation because of financial hardship (40). HADS used to evaluate patients’ anxiety and depression is the most frequently used instrument in Japan, which is utilized to evaluate patients’ anxiety and depression (39). Japanese patients generally felt stressed and anxious suffering from breast cancer and even their parents’ mental conditions were affected as well (41). This had a guiding significance for future policy-making and health medical level assessment of breast cancer in Japan (42).

Relevant medical departments are recommended to make a patient-centered questionnaire with empathy and compassion according to different social environments and national medical levels (43). Meanwhile, we should take into account the difficulties of the elderly in answering questionnaires, which suggests simplifying the questionnaires or formulating different questionnaires for different age groups (44). When selecting which PRO instruments to use, balancing clinicians’ and patients’ preferences should be taken into consideration. Considering that clinicians lacked the knowledge of how to effectively use PRO data in clinical diagnosis and treatment, training concerning PROs should be encouraged to be included in regular medical education curricula (45). Real patient cases and problem-based learning with audio/video clips proved to be the most successful and efficient methods of instruction, enabling physicians to understand how to utilize the PRO instruments and consult the PRO data. Meanwhile, clinical researchers and clinicians should be encouraged to cooperate aiming to formulate patient-centered treatment and care (45).

## 5 Conclusions

The disease burden of female breast cancer is severe and varies greatly throughout countries and regions, while the application of PROs in clinical trials remained noteworthy. It is highly recommended to establish health data on female breast cancer across all countries and territories to raise more awareness about preventive measures and policy-making for female breast cancer to reduce future burdens. Collaboration between clinical researchers and clinicians is encouraged to better estimate the global health impact of female breast cancer, providing valuable evidence to improve health policies and reduce inequalities.

## Data Availability

The original contributions presented in the study are included in the article/**Supplementary Material**. Further inquiries can be directed to the corresponding authors.
