# Supplementary material for: Global burden of breast cancer and application of patient-reported outcomes in clinical trials: a systematic analysis based on the global burden of disease study 2021 and WHO international clinical trial register database
Source: Front Oncol. 2025 Jun 12;15:1557080. doi: 10.3389/fonc.2025.1557080 (PMC12198119; doi:10.3389/fonc.2025.1557080)
Supplement: Supplementary file 1 [file DataSheet1.pdf]

Supplementary Material

eFigure 1. Age standardised point incidence and prevalence of female breast cancer per 100 000 population in 2021, by country.

eFigure 2. Trial exclusion and classification criteria

eFigure 3. Distribution of trials involving PROs in different countries

PRO instruments used in clinical trials

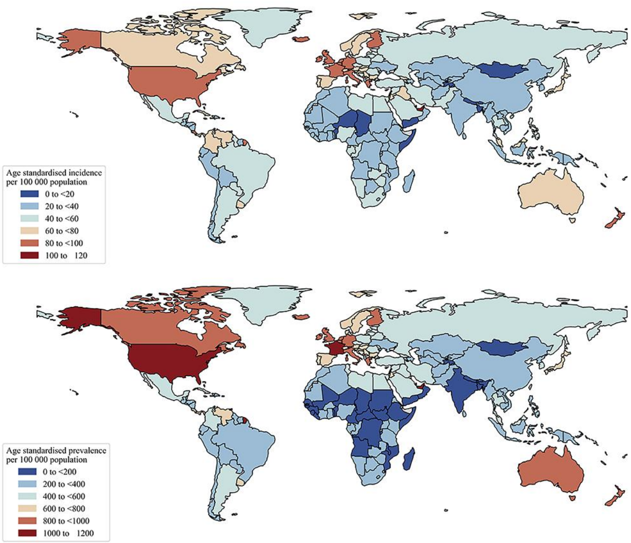

eFigure 1. Age standardised point incidence and prevalence of female breast cancer per 100 000 population in 2021, by country.

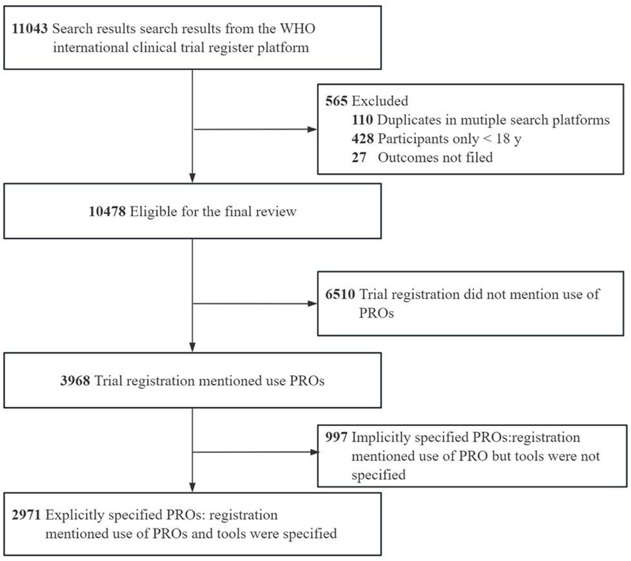

eFigure 2. Trial exclusion and classification criteria

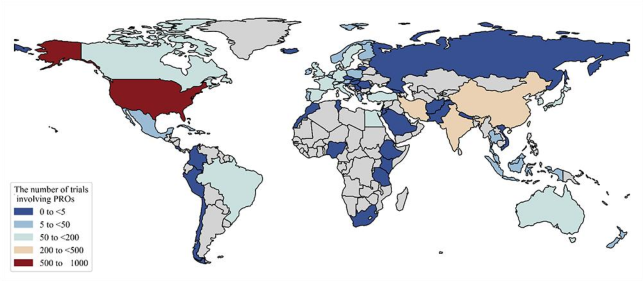

eFigure 3. Distribution of trials involving PROs in different countries

PRO instruments used in clinical trials
